# Successful Outcome of Phytostabilization in Cr(VI) Contaminated Soils Amended with Alkalizing Additives

**DOI:** 10.3390/ijerph17176073

**Published:** 2020-08-20

**Authors:** Maja Radziemska, Agnieszka Bęś, Zygmunt M. Gusiatin, Łukasz Sikorski, Martin Brtnicky, Grzegorz Majewski, Ernesta Liniauskienė, Václav Pecina, Rahul Datta, Ayla Bilgin, Zbigniew Mazur

**Affiliations:** 1 Institute of Environmental Engineering, Warsaw University of Life Sciences, Nowoursynowska 159, 02-776 Warsaw, Poland; grzegorz_majewski@sggw.edu.pl; 2 Faculty of Environmental Management and Agriculture, University of Warmia and Mazury in Olsztyn, Pl. Łódzki 4, 10-727 Olsztyn, Poland; agnieszka.bes@uwm.edu.pl (A.B.); lukasz.sikorski@uwm.edu.pl (Ł.S.); zbigniew.mazur@uwm.edu.pl (Z.M.); 3 Faculty of Geoengineering, University of Warmia and Mazury in Olsztyn, Słoneczna St. 45G, 10 719 Olsztyn, Poland; mariusz.gusiatin@uwm.edu.pl; 4 Department of Agrochemistry, Soil Science, Microbiology and Plant Nutrition, Faculty of AgriSciences, Mendel University in Brno, Zemedelska1, 61300 Brno, Czech Republic; martin.brtnicky@seznam.cz (M.B.); vaclav.pecina@mendelu.cz (V.P.); rahul.datta@mendelu.cz (R.D.); 5 Institute of Chemistry and Technology of Environmental Protection, Brno University of Technology, Faculty of Chemistry, Purkynova 118, 62100 Brno, Czech Republic; 6 Department of Geology and Pedology, Faculty of Forestry and Wood Technology, Mendel University in Brno, Zemedelska 3, 613 00 Brno, Czech Republic; 7 Kaunas Forestry and Environmental Engineering, University of Applied Sciences, Liepu str. 1, Girionys, LT-53101 Kaunas reg., Lithuania; e.liniauskiene@kmaik.lm.lt; 8 Faculty of Engineering, Artvin Coruh University, Seyitler Campus, 08000 Artvin, Turkey; ayla.bilgin@artvin.edu.tr

**Keywords:** soil contamination, soil remediation, immobilizing amendments, risk minimization, *Festuca rubra* L.

## Abstract

This study analysed the effect of three alkalizing soil amendments (limestone, dolomite chalcedonite) on aided phytostabilization with *Festuca rubra* L. depending on the hexavalent chromium (Cr(VI)) level in contaminated soil. Four different levels of Cr(VI) were added to the soil (0, 50, 100 and 150 mg/kg). The Cr contents in the plant roots and above-ground parts and the soil (total and extracted Cr by 0.01 M CaCl_2_) were determined with flame atomic absorption spectrometry. The phytotoxicity of the soil was also determined. Soil amended with chalcedonite significantly increased *F. rubra* biomass. Chalcedonite and limestone favored a considerable accumulation of Cr in the roots. The application of dolomite and limestone to soil contaminated with Cr(VI) contributed to a significant increase in pH values and was found to be the most effective in reducing total Cr and CaCl_2_-extracted Cr contents from the soil. *F. rubra* in combination with a chalcedonite amendment appears to be a promising solution for phytostabilization of Cr(VI)-contaminated areas. The use of this model can contribute to reducing human exposure to Cr(VI) and its associated health risks.

## 1. Introduction

The issue of soil contamination with chromium (Cr) compounds afflicts various regions all over the world [1]. No risk of the global natural environment contamination with Cr compounds has yet been observed. However, when emitted to the atmosphere, soil and water locally, it can be excessively present in biogeochemical circulation [2]. Cr compounds in the environment naturally originate from rock and soil erosion, aerosol deposition and volcano eruptions [3]. Cr is a component of mineral deposits, with chromite ore (FeCr_2_O_4_) being the only one of industrial importance. Among the other Cr compounds, only two occur in small amounts as minerals in the natural environment: lead chromate (PbCrO_4_—crocoite) and sodium dichromate (Na_2_Cr_2_O_7_) [4]. Much larger amounts of Cr are released to the soil from anthropogenic sources as this element is used in many branches of industry [5].

Cr compounds are applied in a variety of industries, e.g., galvanizing processes for protective and ornamental chrome plating of steel and brass items [6] and as an additive to construction steel. Chromates and dichromates are applied as pigments (e.g., chrome yellow PbCr) in manufacturing mineral paints used in ceramic, textile, plastic and paper industries and as paints [7]. Cr compounds are also used as corrosion inhibitors, as polymerization catalysts, as oxidizers in organic synthesis, as raw materials in perfume production and in making inks, as wood preservatives and as light-sensitive materials in photography [8]. Chromite (FeCr_2_) is applied mainly in the production of fireproof materials (fireproof bricks and cement) [9]. Ferrochrome and other compounds, mainly chromates and dichromates are produced from FeCr_2_. Cr(VI) is present during ore processing and the production of sodium and potassium chromate and dichromate, ammonium dichromate, Cr oxide, pigment production (lead and zinc chromate), dyeing textiles (Cr oxide, Cr sulfate) stainless steel and in tanning (Cr_2_[SO_4_]_3_), glass production (Cr oxides) and galvanization (CrO_3_) [10].

However, all Cr(VI) compounds are classified as carcinogenic [11,12]. People are exposed to Cr(VI) compounds present in contaminated potable water, air and soil. Factors that can affect the toxicity of Cr(VI) compounds include bioavailability, oxidative properties and solubility [12]. Even small amounts of Cr(VI) compounds can be harmful to human health [13]. Contact with Cr-containing materials often results in allergic reactions [14].

Soil is an important part of the natural environment and, as such, it should be covered by special protection [15]. Deterioration of the soil quality is largely caused by the ambient concentration of industrial pollutants containing toxic substances, including Cr(VI) compounds [16]. Soil Cr concentration can vary widely, from 1 up to 3000 mg/kg [17]. In soil, highly toxic Cr(VI) is more soluble and more mobile than essential Cr(III). Thus, soil contamination with Cr is dangerous because of the risk of it being taken up by crops grown on such soils, groundwater infiltration and the contamination of potable water intakes [18]. Of all the natural environment components, contamination with Cr is the most persistent in soil. It is associated with metal adsorption on humic colloids and silty minerals [19].

According to current knowledge, phytostabilization is one of the biological techniques of decreasing the health risks of contaminated soil [20,21]. Owing to its high effectiveness and non-invasiveness, it is increasingly often chosen as an effective method for the immobilization of contaminants in the near-surface soil layers [22]. Vegetation (especially the root zone) plays an important role in the stabilization of degraded land. It stabilizes contaminants, including heavy metals [23]. Numerous studies have been performed in terms of tolerance, uptake and accumulation of Cr by several plant species to evaluate the potential of these species for Cr phytoextraction [24,25]. Research on aided phytostabilization of Cr in contaminated soil, in contrast to conventional heavy metals such as Cd, Cu, Zn or Pb, is not so common. Plants used for phytostabilization should immobilize heavy metals in roots (or soil) rather than transport them up to the above-ground parts as they can be further mobilized in the food chain [26]. Until now, plants used for Cr phytostabilization in soil include: plant rose [27], Indian mustard (*Brassica juncea* L.) [28], *Cynodon dactylon*, *Chloris virgata* and *Desmostachya bipinnata* [29]. Among the different plant species, *Festuca rubra* L. has great potential for the immobilization of conventional heavy metals or their fixing in the rhizosphere to reduce metal transport to above-ground parts [30,31]. Since the role of *F. rubra* in phytostabilization of Cr in soil is poorly recognized, it is reasonable to examine the effect of *F. rubra* on the phytostabilization of Cr, especially when it occurs in the soil at different concentrations.

In a relatively new approach to the phytostabilization process, various process additives have been used to enhance phytostabilization (so-called “aided phytostabilization”) [32]. This approach benefits from available natural immobilizing materials (both mineral and organic) and the effects of soil purification. In the present study, natural alkalizing amendments, such as limestone, dolomite and chalcedonite were used as additives aiding the Cr(VI) immobilization processes in acidic soil. The availability of mobile heavy metal species in the soil solution is increased in highly acidic and acidic soils [33]. This is associated with an increase in the solubility of these element compounds and a decrease in their adsorption on soil colloids when the soil pH is low [34]. Therefore, the additives proposed by the authors can be used in the remediation of soil in which the acceptable levels of heavy metal pollution are exceeded because of the alkalizing properties. The novelty of the experiment lies in using new soil amendments, which have not been analysed for an effect of different Cr concentrations on its phytostabilization.

The main objectives of this study were to compare the effectiveness of natural alkalizing amendments in the phytostabilization of Cr with *Festuca rubra* L. and to assess the effect of different Cr concentrations on its phytostabilization. For this purpose, the above-ground biomass of the test plant was determined, and the Cr content was examined in its various organs (above-ground parts and roots). The degree of Cr immobilization in the soil was calculated by determining the total concentration of Cr and the CaCl_2-_extractable Cr. Moreover, an assessment of the phytotoxicity of soil exposed to Cr(VI) compounds was made with the use of *Sinapsis alba* L. and effective concentrations (EC_10_, EC_20_, and EC_50_) were determined.

## 2. Materials and Methods

### 2.1. Plant Experiment

The impact of Cr(VI) at various levels of concentration and amendment of limestone, dolomite and chalcedonite on aided phytostabilization was examined. Pot experiments were performed in a greenhouse (natural daylight, 20–25 °C, 60–70% of humidity). The mass of soil in each polyethylene pot was 3.0 kg. For phytostabilization experiments, the surface soil samples (0–30 cm) from a non-contaminated, agricultural area (northern Poland) were collected. The soil was sandy in texture (86.6% sand, 11.2% silt, 2.2% clay) with acidic pH (5.81). The cation exchange capacity was 94.2 mmol/kg, whereas organic carbon amounted to 0.64%. The nutrient contents in the soil were as follows: total N 0.1%, extractable P 43.2 mg/kg, extractable K 8.72 mg/kg, extractable Mg 31.2 mg/kg.

The soil after air-drying was crushed and sieved to obtain a particle size of ≤ 2 mm. It was then spiked with aqueous solutions of K_2_Cr_2_O_7_ as a source of Cr(VI) to obtain four different metal concentration, i.e., 0 (control), 50, 100 and 150 mg/kg. Before phytostabilization, the soil was fertilized with minerals (N-26%, K_2_O-26%, Cu-0.025%, Mo-0.20%, B-0.013%, Mn-0.25% and Fe-0.05%).

Natural alkalizing amendments (limestone, dolomite, and chalcedonite) were mixed with the spiked and fertilized soil at a dosage of 3.0% (each one). Each treatment was replicated three times. The soil samples were carefully mixed and left for three weeks for stabilization under natural conditions. Seeds of *Festuca rubra* L. cv. Dark were then sown (5 g per each pot) and they germinated six days after sowing. Demineralized water was used for plant watering every other day. The amount of added water corresponded to 60% of the maximum water holding capacity of the soil. The experiment was finished after ca. 47 days after seed sowing. The harvested plants were weighed and then separated into above-ground parts and roots.

### 2.2. Soil Amendments

**Figure 1:**
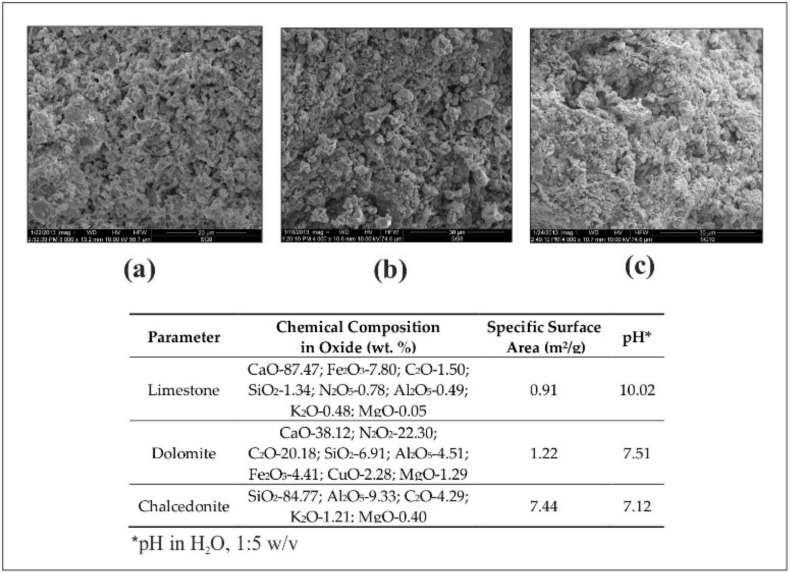
Scanning electron microscope (SEM) images of limestone (**a**), dolomite (**b**), chalcedonite (**c**).

Readily available alkalizing amendments were used in the experiment: limestone, dolomite, and chalcedonite. Figure 1 presents the amendments used (as scanning electron microscope (SEM) images) and their physico-chemical characteristics. Limestone was obtained from the PG Silesia company (Czechowice-Dziedzice, Poland); dolomite from the Dolomite Mine company (Sandomierz, Poland); and chalcedonite from the Chalcedon Poland company in Inowłódz.

### 2.3. Soil Analytical Methods

Soil samples were characterized for: pH (1:5 *w*/*v* suspension in distilled water using a pH meter EA940, Orion, IL, USA), total N (Kjeldahl method [35]), total organic carbon (TOC) (dichromate oxidation of samples followed by titration with ferrous ammonium sulfate [36]), available P (colorimetrically with vanadium-molybdenum method [37]), available K (atomic emission spectrometry) and available Mg (flame atomic absorption spectrometry, FAAS [38]). Total Cr was determined with FAAS using a SpectrAA 280FS spectrometer (VARIAN, Mulgrave, Australia). Before FAAS analyses, soil samples were digested in a mixture of concentrated HCl and HNO_3_ (at 3:1 ratio) in a microwave oven (MARSXpress, CEM Corporation, Matthews, NC, USA) [39]. The samples were then filtered and analyzed for Cr. The accuracy of Cr analysis by FAAS was validated with the CRM 142R reference material.

### 2.4. Plant Chemical Analyses

After the experiment was completed, the plants were removed from the pots to avoid damaging their above-ground and root parts. Harvested *F. rubra* biomass was washed several times with de-ionized water to remove any soil particles and then dried for two weeks at room temperature. Finally, the biomass was homogenized to a fine powder using an analytical mill (Retsch type ZM300, Hann, Germany) and kept at room temperature in closed containers. The dry biomass of roots and shoots was recorded by their drying at 55 °C to a stable weight. The samples of dried and shredded roots and shoots were mineralized in nitric acid (65% *w*/*w*, Chempur, Piekary Śląskie, Poland) and hydrogen peroxide (30% *w*/*w*, Merck, Darmstadt, Germany) in a microwave oven (MARSXpress, CEM Corporation, Matthews, NC, USA). The filtered samples were analyzed for Cr with FAAS using a SpectrAA 280FS spectrometer (VARIAN, Mulgrave, Australia).

### 2.5. Phytotoxicity Analysis

The Cr(VI) toxicity assessment was performed with the germination and early plant growth test (Phytotoxkit FTM) using white mustard (*Sinapsis alba* L.) seeds. The phytotoxicity of Cr(VI) was expressed with the seed germination index, commonly applied in a phytotoxicity assessment. The tests were performed on soils contaminated with Cr (VI) at the following rates: 0, 50, 100 and 150 mg/kg of soil. Soil with no additives and soil with additives (limestone, dolomite, chalcedonite) were tested. A standard OECD soil was used as a control. The test was conducted before *F. rubra* was sown and after the pot experiment was completed. All of the tests were conducted in three replicates. Ten seeds of *S. alba* germinated in special plastic plates on soil covered with filter paper. The plants under study were exposed to the contaminants for 72 h. The plates were incubated in darkness in a thermostatic cabinet (temperature 25 °C). After the set time, the germinated seeds were counted and the root length was measured (Image Tool 3.0 for Windows; UTHSCSA, San Antonio, TX, USA). The germination inhibition (GI) and root growth inhibition (RI) were calculated from the following formula:

(1) $$\mathrm{RI}\;\left(\mathrm{GI}\right)=\;\frac{A-B}{A}\;\times \;100$$

where:

A—seed germination and root length in control (OECD soil),

B—seed germination and root length in a soil sample under examination (soil contaminated with Cr(VI) with and without additives).

Effective concentration (ECx) data were analyzed with a selected regression model to calculate the concentrations at 10, 20 and 50 response levels.

### 2.6. Accumulation Evaluation

The bioaccumulation coefficient (BCF) and translocation factor (TF) were used to analyze the Cr accumulation in the roots of *F. rubra* and the Cr translocation to above-ground parts of the plant and was calculated as follows: BCF = Cr concentration in roots/Cr concentration in soil, TF = Cr concentration in above-ground parts/Cr concentration in roots.

### 2.7. Statistical Analysis

Statistica 13.3 software (San Diego, CA, USA) was used for processing the experimental data. To test the normality of data distribution, the Shapiro–Wilk test was used, and Levene’s test was used to test the homogeneity of variance. A one-way ANOVA and Tukey’s test were applied to determine significant differences (*p* < 0.05) between phytostabilization treatments. The results were also subjected to Principal Component Analysis (PCA) in the XLStat program (Addinsoft, Paris, France).

## 3. Results and Discussion

### 3.1. Effect of Cr(VI) and Soil Amendments on the Biomass of F. rubra

**Figure 2:**
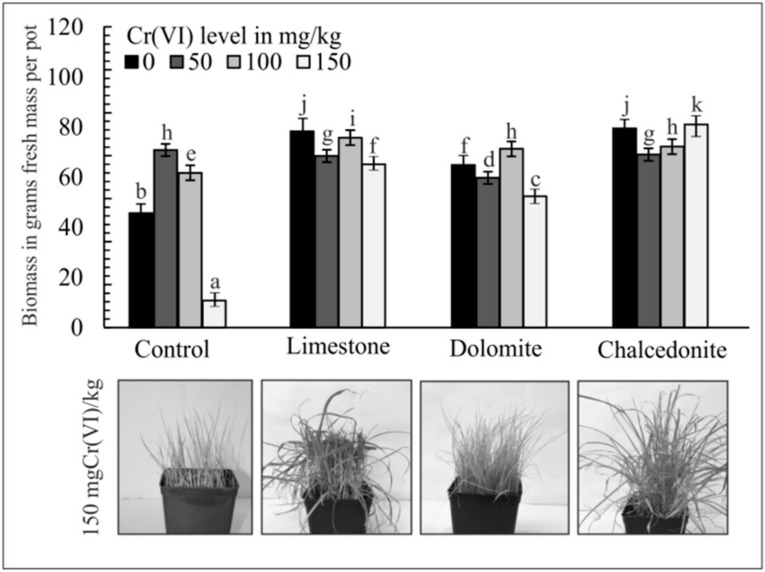
Effect of Cr(VI) and soil amendments (3% *w*/*w*) on the above-ground biomass of *F. rubra*. Error bars are ± standard error (*n* = 3). For a given Cr(VI) level, different letters indicate significant differences between treatments (*p* < 0.05).

Plant response to Cr depends on its concentration and the species in which it is present in the soil [40]. Symptoms of Cr toxicity in plants mainly include water balance disorders, root damage and biomass reduction [41]. Moreover, the visible effects of high contents of Cr include chlorosis and accelerated plant wilting. Plants that are highly sensitive to Cr show these symptoms at concentrations as low as 2 mg/kg [42]. The effects of soil amendments on the biomass of *F. rubra* grown in Cr(VI)-contaminated soils are shown in Figure 2. In pots with no soil amendments, the above-ground parts of *F. rubra* were affected by the highest Cr(VI) concentration, which is demonstrated by a significantly lower yield compared to pots with the amendments. This relationship was corroborated by Seleiman et al. [43] who found that Cr negatively affected biomass and yield of *Triticum aestivum* L., and Shankera et al. [44] and Golovatyj et al. [45], who reported that the above-ground biomass yield of *Zea mayze* L. and *Hordeum vulgare* L. decreased significantly in the presence of Cr compounds in soil. Among the soil amendments applied in this experiment, limestone increased the mean yield (for all the variants Cr(VI) level) of the above-ground parts of *F. rubra* by 48% and chalcedonite by 45% as compared to the control. The lowest above-ground biomass was found for the experiment with dolomite. Wyszkowski and Radziemska [3,46] reported that the application of alkaline soil amendment increased the mean yield of plants (*Avena sativa* L., *Hordeum vulgare* L. and *Zea mayze* L.) grown on Cr(VI)-polluted soil.

### 3.2. Cr Accumulation in F. rubra

**Figure 3:**
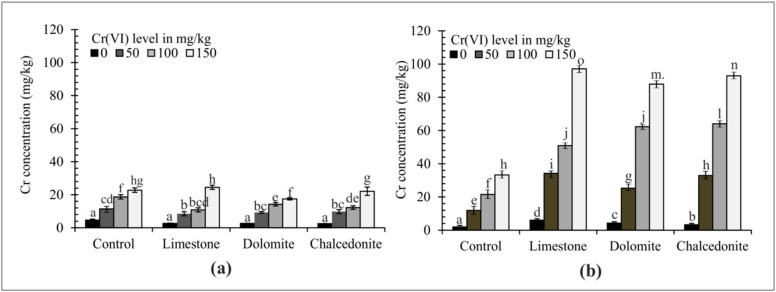
The effect of soil amendments (3% *w*/*w*) on Cr accumulation in the above-ground part (**a**) and roots (**b**) of *F. rubra*. Error bars are ± standard error (*n* = 3). For a given Cr(VI) level, different letters indicate significant differences between treatments (*p* < 0.05).

Physicochemical soil properties, such as pH, texture and humus content, considerably affect the degree of oxidation of Cr compounds [47] which, in consequence, affects their toxic effect on plants. Cr(VI) is very harmful to plants—the Cr_2_O_7_^2−^ compound is highly toxic, while Cr^3+^ contamination at the same concentration causes no damage [48]. The concentration of Cr in the roots and above-ground parts of *F. rubra* correlated with the Cr(VI) concentration and all amendments added to the soil (Figure 3). The Cr content was higher in the roots than in the above-ground parts of the test plant. This relationship is corroborated by the findings of a study by Ram et al. [49], in which the Cr concentration and accumulation in roots of hybrid Napier grass was significantly higher than in the above-ground parts. Soil contamination with Cr(VI) at 150 mg/kg resulted in the highest Cr accumulation in the analyzed plant. The alkalizing mineral amendments, which can increase soil sorptive capacity, can also reduce the content of heavy metals in the soil available to plants [48,50].

For parameters such as BCF and TF, which determine the plant effectiveness for phytostabilization, according to Mendez and Maier [51], BCF should be higher than 1 and TF should be lower than 1. The species in which heavy metals are present in soil are also important soil parameters, affecting the translocation process [52]. The highest mean BCF for *F. rubra* was observed under the chalcedonite treatment, whereas the lowest TF was observed under the limestone treatment. The highest BCF values were observed following limestone, dolomite, and chalcedonite application, when the soil was contaminated with Cr(VI) at 50 mg/kg.

### 3.3. Phytotoxicity

**Figure 4:**
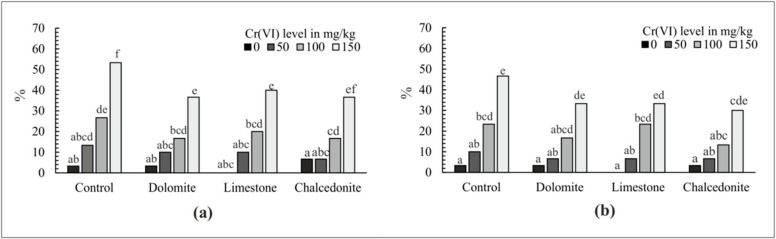
Germination inhibition index for *S. alba*—GI (%); (**a**) before phytostabilization started; (**b**) after phytostabilization was completed. For a given Cr(VI) level, different letters above the columns indicated a significant difference at *p* < 0.05. Error bars are ± standard error (*n* = 3).

**Figure 5:**
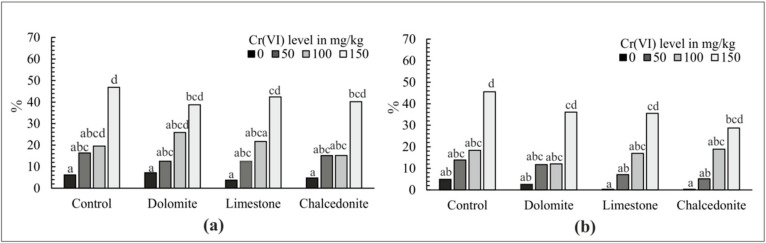
Root growth inhibition index for *S. alba*—RI (%); (**a**) before phytostabilization started; (**b**) after phytostabilization was completed. For a given Cr(VI) level, different letters above the columns indicated a significant difference at *p* < 0.05. Error bars are ± standard error (*n* = 3).

The phytotoxicity test, performed on the soil before the phytostabilization experiment was set up, showed increasingly high GI in soil with no additives and increasing Cr(VI) concentrations (Figure 4a). The analysis of root growth produced similar observations (Figure 5a). The strongest inhibitory effect on the germination potential and the root length in *S. alba* was observed at the Cr(VI) rate of 150 mg/kg of soil. The lowest RI index was observed in soil contaminated with Cr(VI) at 0 and 50 mg/kg soil—6.18% and 16.4%, respectively. A similar effect was observed in the soil with the additives. The highest indices: of GI and RI—37.8% and 40.5%, respectively—were noted for the Cr(VI) rate of 150 mg/kg of soil, regardless of the additive applied. However, these values were lower than in the soil with no additives, which indicates that alkalizing additives alleviate the toxic effect of Cr. These data indicate a positive response of the test plants to the applied additives, which manifests itself by an increase in the produced biomass [53]. Growth and germination inhibition were observed at the rates of 50 and 100 mg/kg of soil. The differences were small, but statistically significant. A comparison of the findings with those for soil with no additives demonstrated that the indices at the doses of 50 and 100 mg/kg of soil were much lower (Figure 4a and Figure 5a). The dose of 50 mg/kg of soil proved to be the least toxic to plants, which was similar to soil with no additives. A comparison of the additives showed that chalcedonite had the greatest stimulating effect on germination and root growth, followed by limestone and dolomite, regardless of the contamination level.

The phytotoxicity test performed on soil samples after the phytostabilization indicated that soil contaminated with Cr(VI) with no additives was still highly toxic to *S. alba*. However, the application of additives decreased the GI index and the RI index considerably. Chalcedonite proved to be the most effective additive, as in the first test (Figure 4b and Figure 5b).

**Figure 6:**
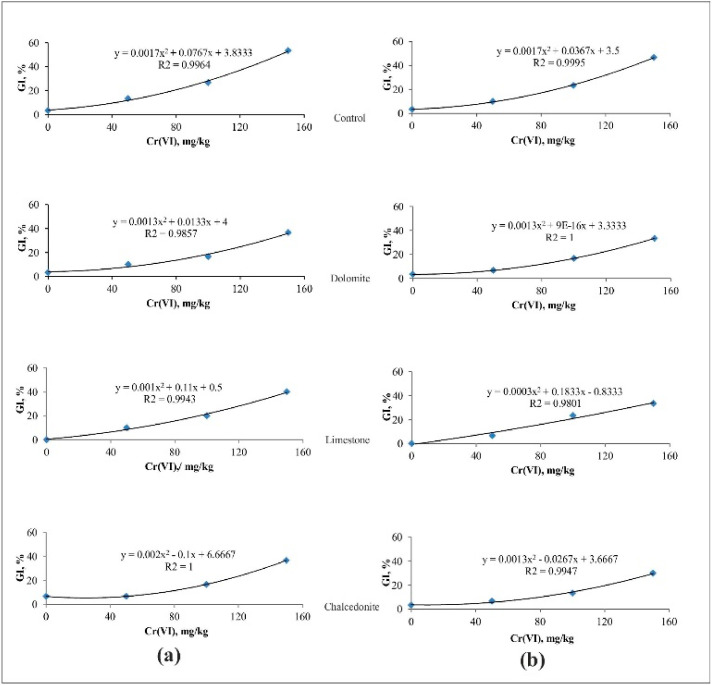
The effect of Cr(VI) on inhibition in germination (GI) of *S. alba* (**a**) before phytostabilization, (**b**) after phytostabilization.

The lowest observed effective concentration is the lowest concentration of a contaminant which reduces the measured response by more than 20% [54]. It was found that the 20% of GI in *S. alba* in the control soil was caused by its contamination with Cr(VI) at 77.3 mg/kg. Mixing the same soil with dolomite, limestone and chalcedonite decreased the phytotoxicity of Cr(VI). This effect was the most clearly visible in soil with chalcedonite, where EC_20_ = 114 mg/kg. The response of *S. alba* in the control soil as 50% GI (EC_50_) was observed at a Cr(VI) concentration of 146 mg/kg. However, in the experiment variants using mixtures with dolomite, limestone and chalcedonite, the germination was inhibited by 37.4, 21.4 and 28.6 mg/kg at a higher concentration of Cr (Table 1, Figure 6).

**Figure 7:**
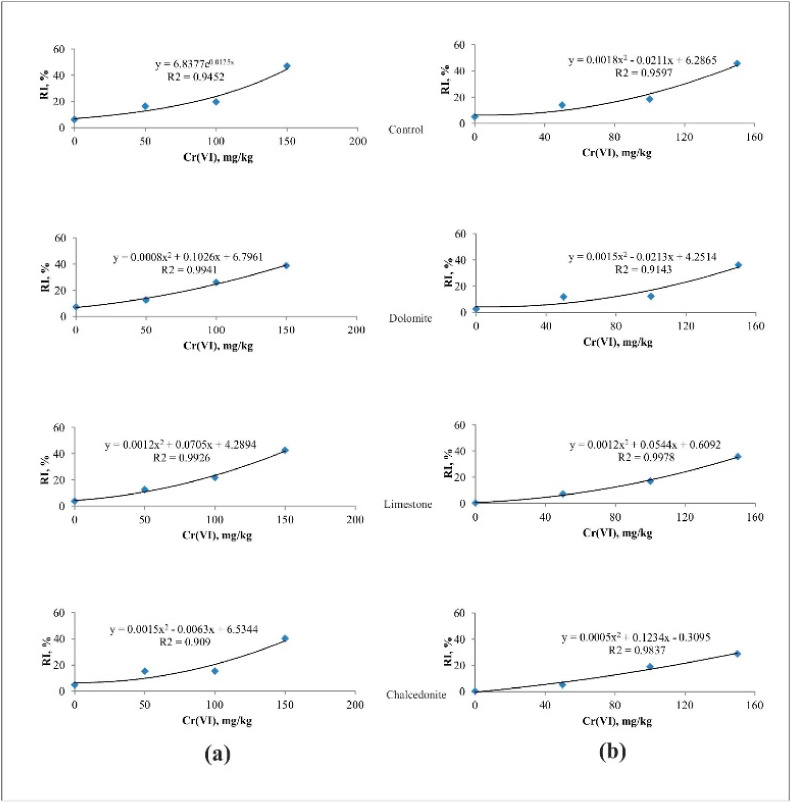
The effect of Cr(VI) on growth inhibition of roots (RI) of *S. alba* (**a**) before phytostabilization, (**b**) after phytostabilization.

After six weeks of growing *F. rubra*, a three-day Phytotoxkit test was performed on each soil under test. Germination inhibition in *S. alba* caused by Cr(VI) was lower both in the control soil and in soil with amendments than before sowing *F. rubra*. Cr was the most toxic in the control soil (mean EC = 96.5 mg/kg Cr(VI), and the least toxic in soil with dolomite (mean EC = 136 mg/kg of Cr) (Table 1, Figure 7).

**Figure 8:**
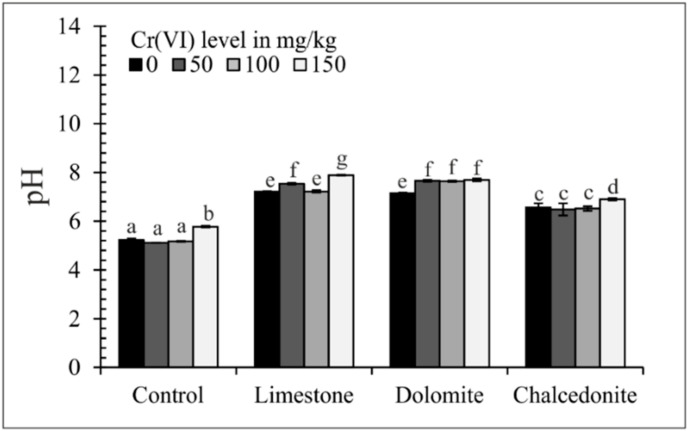
Soil pH. Error bars ± standard error (*n* = 3). For a given Cr(VI) level, different letters indicate significant differences between treatments (ANOVA# followed by Tukey’s HSD test, *p* < 0.05).

Increasing Cr(VI) concentrations not only inhibited *S. alba* germination but also the growth of its roots (RI). The effective concentration of Cr(VI) in the control soil before growing *F. rubra*: EC_10_, EC_20_ and EC_50_ inhibiting the growth of *S. alba* roots were 28.1, 86.5, 154 mg/kg, respectively. However, the effective concentrations in the least toxic soil (with an addition of chalcedonite) were, respectively: EC_10_ = 45.5, EC_20_ = 99.0 and EC_50_ = 172.3 mg/kg of Cr. Growing *F. rubra* for six weeks improved the quality of all the soils with additives. In each soil contaminated with the same Cr(VI) doses, the roots of *S. alba* grew more intensively than before sowing *F. rubra*. The highest growth of *S. alba* roots was observed in soil with chalcedonite (mean EC = 123 mg/kg Cr). The highest RI was observed in the control soil (mean EC = 97.6 mg/kg of Cr) (Table 1, Figure 8).

### 3.4. Soil Chemical Properties

The occurrence of Cr compounds in soil depends on the pH and redox potential [55]. The sorption of Cr(III) increases and that of Cr(VI) decreases with increasing soil acidity. An increase in soil acidity accelerates many processes in which such harmful elements as Al and Mn are released from the sorption complex to the soil solution [56]. Therefore, it seems justified to use soil additives which increase the soil pH significantly. The soil pH following the phytostabilization experiment is shown in Figure 8. The greatest increases were 2.15 and 2.22 pH units, with limestone and dolomite added into soil. This was corroborated by Ye et al. [57], who found that an addition of an alkalizing amendment (diatomite) was found to increase the soil pH.

**Figure 9:**
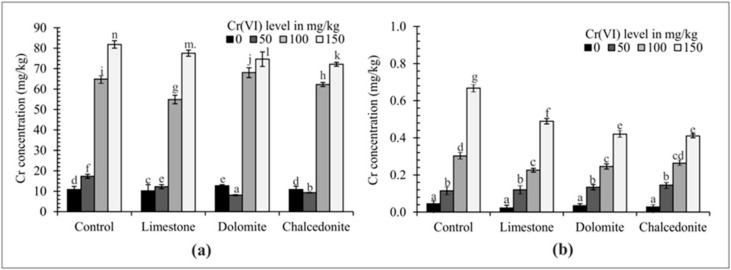
Total (**a**) and CaCl_2_-extractable (**b**) Cr concentrations in the soil after the application of soil amendments (3% *w*/*w*). Error bars are ± standard error (*n* = 3). For a given Cr(VI) level, different letters indicate significant differences between treatments (*p* < 0.05).

The mobility and availability of heavy metals in soil depend on their total concentration and that of exchangeable forms [58]. In the natural environment Cr(VI) compounds are easily reduced by organic matter to Cr(III) compounds. [59]. The total (a) and CaCl_2_-extractable (b) Cr content is shown in Figure 9. The Cr concentration after the phytostabilization experiment was the lowest following the application of limestone and chalcedonite compared to the control pots. CaCl_2_ extraction is a measure of metal availability [30]. The application of alkalizing amendments reduced CaCl_2_-extractable content of Cr in soil, and the most significant reduction was observed under dolomite and chalcedonite treatment.

### 3.5. Statistical Analysis

**Figure 10:**
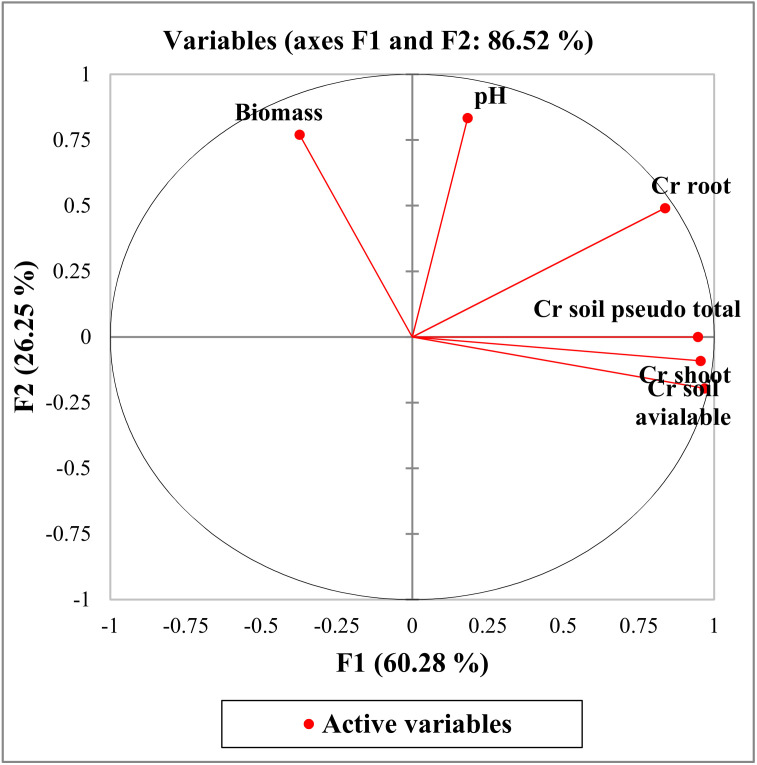
Plot of principal component scores (PCA).

The correlation matrix of data obtained from PCA is shown in Table 2 and Figure 10. There was a significant positive relationship between Cr content in the shoots and in the roots (*r* = 0.757), Cr total content in the soil (*r* = 0.876) and Cr in the soil in an available form (*r* = 0.936). There was also a significant positive correlation between Cr in the root and the soil available forms (*r* = 0.695) and soil pH (*r* = 0.505), respectively. A significant positive correlation was also found between total Cr in the soil and in an available form (*r* = 0.895). There was also a significant negative correlation between *F. rubra* biomass and Cr content in the soil in an available form (*r* = –0.538). The results indicate that the accumulation of Cr in plant tissues depended on total Cr concentration, its available form and soil pH.

Suitability for a data factor analysis was tested by Kaiser–Meyer–Olkin (KMO) and Barlett Tests. The value of KMO should be greater than 0.5. The data are suitable for the factor analysis as the KMO value gets closer to 1 between 0 and 1 [60,61]. According to the analysis results, the value of KMO = 0.621 is suitable to be used in this analysis. As a result of the factor analysis, two factors were identified with eigenvalues greater than 1. These three factors explain 86.5% of the total variance. The first factor explains 60.3% of the total variance and Cr in the shoot and root, total content and available form in the soil have strong positive load values, respectively. The second factor explains 26.3% of the total variance and soil pH and plant biomass have strong positive load values, respectively.

## 4. Conclusions

Based on the present study, it can be concluded that the application of alkalizing amendments such as limestone, dolomite and chalcedonite has a beneficial effect on aided phytostabilization of soil contaminated with Cr(VI) at various doses. This effect is accompanied by increased soil pH, which is the highest following the application of limestone and dolomite. The current results show that an addition of chalcedonite brings about the highest yield of *F. rubra*, the highest concentration of Cr in roots and the highest mean bioconcentration factor. The phytotoxicity test results show that chalcedonite has the greatest stimulating effect on *S. alba* germination and root growth, followed by limestone and dolomite, regardless of the Cr contamination level. The findings present great potential for practical application because of the availability of the amendments under study, their environmental safety and high effectiveness in chromium immobilization. Moreover, they help to recreate vegetation in degraded areas. Using this kind of phytostabilization may contribute to the reduction of Cr(VI) exposure and thus to the reduction of human health risk in Cr-contaminated areas.

## Figures and Tables

**Table 1:** The effect of Cr(VI) on inhibition in germination (GI) and growth inhibition of roots (RI) of *S. alba* growing in control soil and soil supplemented with dolomite, limestone and chalcedonite. EC_10_, EC_20_ and EC_50_ values are expressed in mg/kg.

Effective Concentration mg/kg	Control	Dolomite	Limestone	Chalcedonite
	Inhibition in germination (GI) before remediation			
EC_10_	38.0	58.6	55.3	63.3
EC_20_	77.3	110	96.2	114
EC_50_	146	183	167	174
Mean Effective Concentration	87.0	117	106	117
	Inhibition in germination (GI) after remediation			
EC_10_	47.6	91.9	53.8	68.2
EC_20_	89.6	141	96.9	121
EC_50_	152	173	203	179
Mean Effective Concentration	96.5	136	118	123
	Growth inhibition of roots (RI) before remediation			
EC_10_	28.1	25.0	42.3	45.5
EC_20_	86.5	79.6	89.7	99.0
EC_50_	154	172	161	172
Mean Effective Concentration	89.4	92.2	97.5	106
	Growth inhibition of roots (RI) after remediation			
EC_10_	44.2	65.5	67.1	59.4
EC_20_	95.8	115	110	107
EC_50_	153	182	181	202
Mean Effective Concentration	97.6	121	120	123

**Table 2:** Correlation matrix between soil physico-chemical properties and plant-related parameters.

	Biomass	Cr Shoot	Cr Root	Cr Soil Total	Cr Soil Available	pH
Biomass	1					
Cr shoot	–0.338	1				
Cr root	0.086	0.757	1			
Cr soil total	–0.278	0.876	0.776	1		
Cr soil available	–0.538	0.936	0.695	0.895	1	
pH	0.317	0.023	0.505	0.106	0.055	1
